# Supplementary material for: Spectroscopic and Spectroelectrochemical Studies of Hexapentyloxytriphenylene—A Model Discotic Molecule
Source: Int J Mol Sci. 2023 Apr 8;24(8):6924. doi: 10.3390/ijms24086924 (PMC10139095; doi:10.3390/ijms24086924)
Supplement: Supplementary file 1 [file ijms-24-06924-s001.zip › ijms-2302928-supplementary.pdf]

Supplementary Information

Spectroscopic and Spectroelectrochemical Studies of Hexapentyloxytriphenylene – a Model Discotic Molecule

Piotr Sleczkowski

International Center for Research on Innovative Biobased Materials (ICRI-BioM)-International Research Agenda, Lodz University of Technology, Zeromskiego 116, 90-924 Lodz, Poland; piotr.sleczkowski@p.lodz.pl

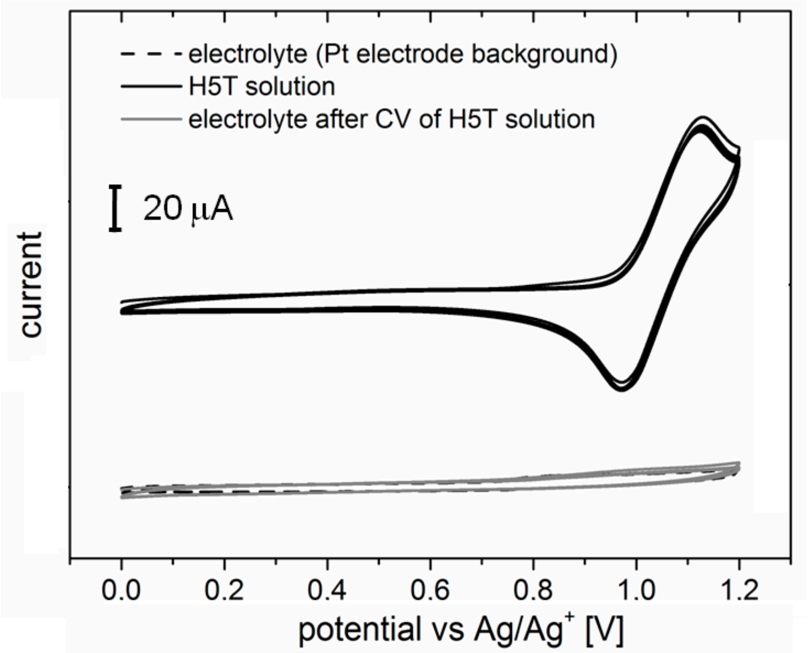

Figure S1. Reversibility of the H5T radical cation formation in the potential window 0.00-1.20 V. A 25-cycle anodic cyclic voltammogram of H5T at 1 × 10^{-3} mol dm^{-3} in a solution of dichloromethane containing 0.2 mol dm^{-3} of Bu_4NBF_4 as the electrolyte (black solid line). Pt electrode in the electrolyte solution before (black dashed line) and after (gray solid line) performing CV of H5T sample, revealing no differences. Scan rate 0.1 V s^{-1}.

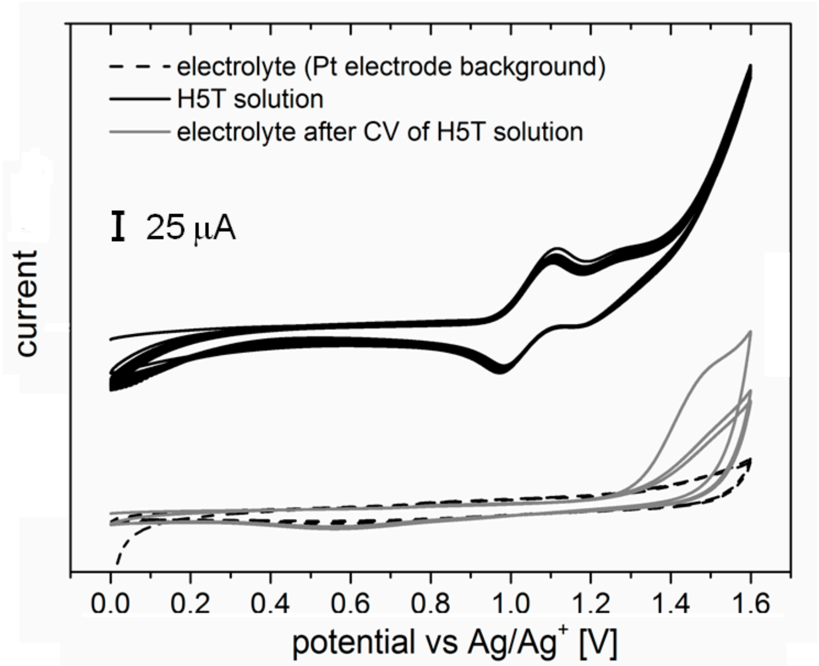

Figure S2. A 25-cycle anodic cyclic voltammogram of H5T at 1×10^{-3} mol dm^{-3} in a solution of dichloromethane containing 0.2 mol dm^{-3} of Bu_4NBF_4 as the electrolyte (black solid line). Pt electrode in the electrolyte solution before (black dashed line) and after (gray solid line) performing CV of H5T sample, showing irreversibility. Scan rate 0.1 V s^{-1}.

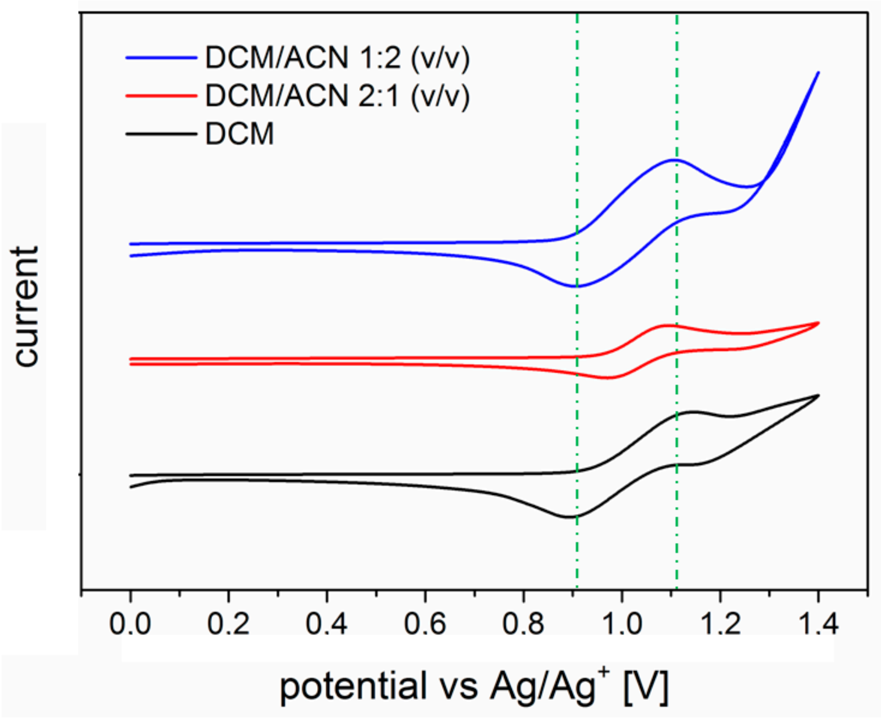

Figure S3. Anodic cyclic voltammograms of H5T solutions in DCM (black line) and in DCM:acetonitrile solutions with solvent volume ratios of 2:1 and 1:2 (red and blue lines, respectively). The data shows minor influence of acetonitrile content on the oxidation of H5T, with negligible shifts in oxidation wave and the related half-wave potential. H5T concentration equal 1×10^{-3} mol dm^{-3}, 0.2 mol dm^{-3} of Bu_4NBF_4 as the electrolyte. Scan rate 0.1 V s^{-1}.

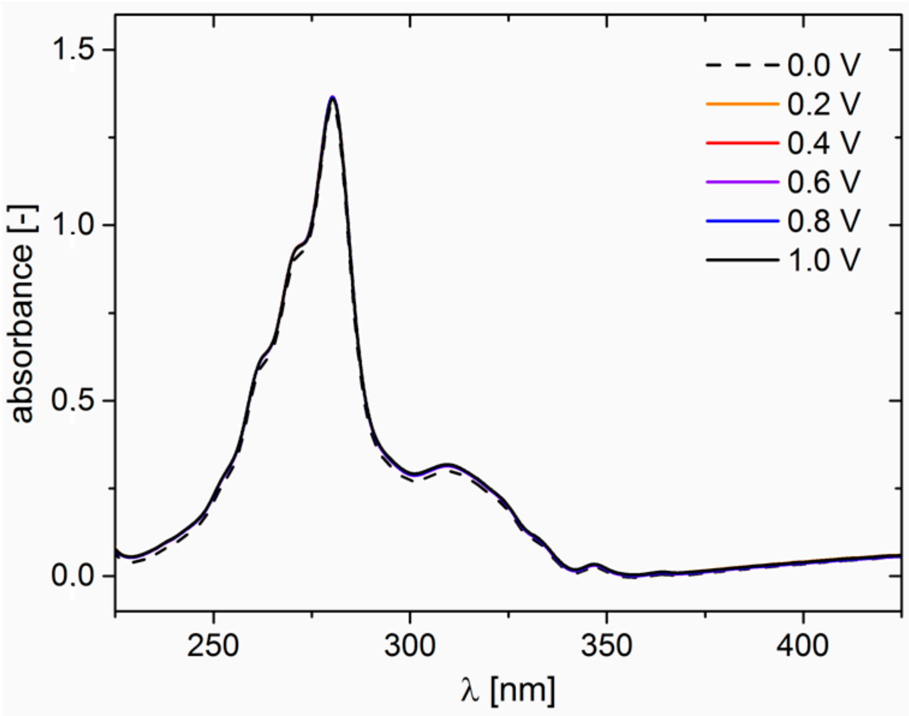

Figure S4. Spectroelectrochemistry of H5T in a solution of dichloromethane containing 0.2 mol dm^{-3} of Bu_4NBF_4 as the electrolyte, revealing no spectral changes for the applied potentials up to 1.0 V.
